# Relationship between mutuality and depression in patients with chronic heart failure and caregivers in China: An actor-partner interdependence model analysis

**DOI:** 10.3389/fpsyg.2022.928311

**Published:** 2022-09-08

**Authors:** Ting Zhou, Jiling Qu, Huiping Sun, Mengxin Xue, Yongbing Liu

**Affiliations:** School of Nursing, Yangzhou University, Yangzhou, China

**Keywords:** actor-partner interdependence model, chronic heart failure, depression, dyads, mutuality

## Abstract

**Background:**

Patients with chronic heart failure and their family caregivers may experience adverse emotional problems, such as depression. Mutuality, which refers to the relationship between caregivers and those they care for, is an important factor affecting depression in the dyads. The purpose of this study was to investigate the relationship between mutuality and depression in patients with CHF and their caregivers in China.

**Methods:**

In this cross-sectional study, we used the Mutuality Scale and the Self-Rating Depression Scale to measure mutuality and depression of patients and caregivers. We used SPSS version 26.0 and AMOS version 21.0 to analyze the data. An APIM was established to analyze the actor-partner effects of patient-caregiver mutuality and depression.

**Results:**

A total of 250 dyads of patients and caregivers were included in the study. There were statistically significant differences in mutuality and depression between CHF patients and caregivers. The 4 dimensions of patients’ mutuality all have the actor effect on depression. There were 3 partner effects of caregivers’ “pleasurable activities”, “shared values”, and “reciprocity” on depression. Regarding caregiver depression, we only found an actor effect of caregivers’ “shared values” on depression.

**Conclusion:**

The relationship between patients and caregivers should be evaluated in the clinical setting, and it is very important to develop intervention measures to improve the adverse emotional problems affecting both patients and their caregivers.

## Introduction

Chronic heart failure (CHF) is a syndrome of cardiac insufficiency caused by various heart diseases, and it is also the end stage of heart disease (Chinese Association of Cardiology Heart Failure Group, 2018). The American Heart Association predicts that 8 million Americans will suffer from HF by 2030 (Virani et al., 2020). The Writing Committee of the Report on Cardiovascular Health and Diseases in China reported that 8.9 million patients suffered from HF in 2019 (The Writing Committee of the Report on Cardiovascular Health and Diseases in China, 2020) and its prevalence continues to rise according to Expert Committee of Chinese Heart Failure Center (Expert Committee of Chinese Heart Failure Center, 2018). The prevalence of CHF in the population of China is 0.9% (Chen et al., 2015). Symptoms of CHF can recur, and patients usually need to be hospitalized more than once. In China, the one-year readmission rate for patients with CHF was 56% in 2018 (Al-Omary et al., 2018). CHF is characterized by high prevalence, high readmission rate, and high medical expense (Benjamin et al., 2019), and it causes great distress and burden to patients and their families (Liu et al., 2020).

The high prevalence, mortality, and readmission rates of CHF lead to negative emotions, such as depression in both patients and their caregivers. People with depression are more likely to develop HF and depression increases the probability of death in patients with HF (Adelborg et al., 2016; Chobufo et al., 2020). Caregivers with HF also have a higher prevalence of depression, and caregiver depression is associated with an increased risk of poor quality of life (Clements et al., 2020). In addition, depression in patients with HF has been shown to be associated with poorer emotional health among caregivers (Chung et al., 2016).

Mutuality refers to the relationship between caregivers and those they care for, and it can reflect the positive quality of the relationship between patients and caregivers (Archbold et al., 1990). Mutuality is a feeling of intimacy, connection, and understanding of others. It is a feeling of sharing, participating, and satisfying others (Liu, 2006). It represents the emotional connection between patients and caregivers. Mutuality is composed of 4 dimensions, which reflect love and affection, pleasurable activities, shared values, and reciprocity. Some scholars have studied the relationship between patients with HF and their main family caregivers in the United States and Italy, and the results showed that mutuality was above the average level and that patients perceived a higher mutuality than did their main family caregivers (Hooker et al., 2018; Vellone et al., 2018; Dellafiore et al., 2019). Similar results were found in a Chinese study (Zhang et al., 2020). In patients with cancer, higher levels of patient-caregiver dyads interactions were associated with lower anxiety and depression in patients (Schumacher et al., 2007). Similar findings were reported for patients with dementia (Shyu et al., 2010) and coronary heart disease (Halm and Bakas, 2007). Studies showed that the mutuality was associated with positive emotions in CHF patients and caregivers in China (Gong et al., 2021; Peng et al., 2021). Patients and family caregivers are interdependent interactive systems when facing the disease together, in which they can perceive, assess, communicate and cope with stress. The dyads who perceive better mutuality may adopt positive coping behaviors, such as positive and timely communication between the dyads, which can reduce conflict and negative emotions (Li et al., 2022). To date, the relationship between mutuality and depression in CHF patients and caregivers is rarely reported. The question of how does mutuality between patients and caregivers impact dyads’ emotions remains.

There is limited information about whether patients’ and caregivers’ mutuality are related to their depression. In addition, most previous studies examined the relationship between mutuality and depression at the individual level. Therefore, it is necessary to explore the interaction between patients and caregivers at the dyad level.

Both patients and caregivers are affected by the patients’ illness, and there are also interactions between the dyads. The actor-partner interdependence model (APIM) can be used to examine the relationship between dyadic variables (Lyons and Lee, 2018). The individual dependent variable is not only affected by the individual independent variable but also by the independent variable of others (Kenny and Cook, 1999). The effect of a person’s independent variable on the person’s own dependent variable is called the actor effect. The effect of a person’s independent variable on his/her dyadic partner’s dependent variable called the partner effect. The model takes into account the non-independence of the individual data in the dyadic relationship, and the interdependence of the dyadic data can be controlled using the model. In China, researchers have applied the APIM to study the effect of intimate relationships on quality of life of colorectal cancer patients and their family caregivers (Wu et al., 2020). However, the APIM has not been used to explore the relationship between mutuality and depression in CHF patients and their caregivers. Therefore, the objectives of this study were to compare the level of mutuality and depression between patients with CHF and their caregivers and evaluate whether the depression of patients and caregivers is related to their mutuality.

## Materials and methods

### Design

This cross-sectional study was a secondary analysis from data collected in a study evaluating the effects of mutuality, unmet needs on depression in CHF patient-caregiver dyads. The study was powered to answer the main outcome of the parent study. In order to better understand the effect of mutuality on depression. In this secondary analysis, we also analyzed the effects of four dimensions of mutuality on depression. The Ethics Committee of the School of Nursing, Yangzhou University approved the research protocol (approval number: YZUHL20200013).

### Sample and setting

In the parent study, patients with CHF who were admitted to two grade A general hospitals in Yangzhou, China from March to May 2021 and their family caregivers were investigated. Inclusion criteria for patients were: (1) meeting the diagnostic and grading criteria for CHF set by the New York College of Cardiology and being diagnosed as having CHF with New York Heart Association class ranging from I to IV; diagnosed for ≥ 1 month; (2) 18 years or older; (3) having at least one family caregiver; (4) able to complete the questionnaire in written or oral form; and (5) provided informed consent and voluntary participation. Exclusion criteria for patients were: (1) having other critical diseases, such as malignant tumor, renal failure, or respiratory failure; or (2) cognitive impairment or mental disease. Inclusion criteria for family caregivers were: (1) being the primary caregiver of the patient, who provided free care; (2) 18 years or older; (3) able to complete the questionnaire in written or oral form; and (4) provided informed consent and voluntary participation. Exclusion criteria for family caregivers were: (1) receiving payment for care; (2) having cognitive impairment or mental disease; or (3) having serious physical diseases, such as cancer or severe organ failure.

### Measurements

We used the Mutuality Scale (MS), developed by Archbold (Archbold et al., 1990) in 1990, to explore the relationship between patients and caregivers. The scale contains 15 items included in the dimensions of love and affection, pleasurable activities, shared values, and reciprocity. A Likert 5-level scoring method was used for all items, with 0 indicating not at all and 4 indicating a great deal, and the average scoring method was adopted. Mutuality scores were converted to a standardized score of 0-100 for each domain (converted score = [(actual raw data-0)/(4-0)] × 100). A score of 0 indicates the worse mutuality, and a score of 100 indicates the better mutuality. Higher scores indicate better mutuality, whereas a score <2.5 (converted score <62.5) indicated poor mutuality (Kneeshaw et al., 1999; Zhang et al., 2020; Gong et al., 2021). This scale was introduced to China by Liu in 2006 (Liu, 2006). The total Cronbach’s alpha was 0.94.

We used the Self-Rating Depression Scale (SDS) to evaluate the depression status of patients and caregivers. The scale consists of 20 items, and a Likert 4-level scoring method was used. The scores of the 20 items are summed to obtain the total rough score, which is then multiplied by 1.25 and rounded to obtain the standard score. A higher score indicates greater likelihood of being depressed. Liu et al. (1995) used the Chinese version of the SDS to evaluate 560 people and the Cronbach’s alpha was 0.86. The results of structural validity analysis were in line with the evaluation requirements of the scale, indicating that the SDS has good reliability and validity. The total Cronbach’s alpha was 0.82.

We also collected patients’ demographic information and clinical data (e.g., age, gender, education level, marital status, time of illness, times of hospitalization, New York Heart Association class (NYHA), Charlson Comorbidity Index (CCI), Left Ventricular Ejection Fraction (LVEF), Body Mass Index (BMI), smoking and drinking). Caregivers’ demographic information (age, gender, relationship to patient, education level, marital status, employment, monthly income, total time spent in care, hours per day spent caregiving, current illness) was collected.

### Statistical analysis

Microsoft Excel 2019, IBM Statistical Package for Social Sciences (SPSS) version 26.0 and IBM SPSS AMOS version 21.0 were used to analyze the data. Descriptive statistics were used to describe patient-caregiver dyads mutuality and depression scores. Paired-sample *t* tests were used to compare the scores of each variable between patients and family caregivers. We compared patient-caregiver dyads mutuality and depression scores using radar maps. Radar map is a common graph used for multivariate comparative analysis. In a radar map, each variable has its own numerical axis. These numerical axes have a common center and radiate around this center to form a radar-like image, also like a spider’s web, so it is called radar map or spider’s web (Zhu, 2006). It can clearly reflect the evaluation of the overall development of the individual. Pearson correlation was used to analyze the relationship between patient and caregiver mutuality and depression. We used Amos 21.0 to analyze dyadic data. An APIM was established to analyze the actor-partner effects of patient-caregiver mutuality and depression. The mutuality was taken as independent variable and depression as dependent variable. A separate APIM for each dimension of mutuality was fitted. *P* < 0.05 was considered to be statistically significant.

## Results

### Characteristics of patients with chronic heart failure and their caregivers

A total of 250 dyads of patients and their caregivers were included in this study (Table 1). Most patients were male (55.6%) and married (77.2%). The average age was 70.94 + 12.86 years. The average number of years of illness was more than 4 years, and the average number of hospitalizations was more than one time a year. The education level of the patients is low, with only 23.2% receiving education beyond middle school. Most patients were classified as New York Heart Association class III or IV (46.8% or 41.6%). The LVEF (51.32 ± 15.86) and CCI (4.32 ± 1.64) of patients indicated that patient’s condition was poor. Few of patients smoke (36.0%) and drink alcohol (30.8%). Most of the patients (45.2%) had a body mass index in the normal range.

**TABLE 1:** Patients (*n* = 250) and caregivers (*n* = 250) characteristics.

Characteristics	Patients M ± SD or n (%)	Caregivers M ± SD or n (%)
		
Age	70.94 ± 12.86	60.20 ± 13.31
Gender		
Male	139(55.6)	98(39.2)
Female	111(44.4)	152(60.8)
Education level		
≤ Middle school	192(76.8)	176(70.4)
>Middle school	58(23.2)	74(29.6)
Marital status		
Married	193(77.2)	240(96.0)
Single/divorced/widowed	57(22.8)	10(4.0)
Years of illness (year)	4.12 ± 5.04	
Number of hospitalizations in 1 year	1.88 ± 1.31	
NYHA class		
II	29(11.6)	
III	117(46.8)	
IV	104(41.6)	
LVEF (%)	51.32 ± 15.86	
BMI (kg/m^2^)		
<18.5	17(6.8)	
18.5∼	113(45.2)	
24.0∼	81(32.4)	
≥ 28.0	39(15.6)	
CCI	4.32 ± 1.64	
Smoking	90(36.0)	
Drinking	77(30.8)	
Relationship to patient		
Parent		4(1.6)
Spouse		137(54.8)
Daughter or son		95(38.0)
Friend		2(0.8)
Other		12(4.8)
Job		
Unemployed or retired		88(35.2)
Employed		162(64.8)
Monthly income (CNY)		
<2000		30(12.0)
2000∼		145(58.0)
≥ 5000		75(30.0)
Total time spent in care (year)		
≤ 1		139(55.6)
>1		111(44.4)
Hours per day spent caregiving (hour)		
≤ 2		92(36.8)
>2		158(63.2)
Current illness		
Hypertension		39(15.6)
Diabetes mellitus		12(4.8)
Heart disease		6(2.4)
Others		3(1.2)

NYHA, New York Heart Association; CCI, Charlson Comorbidity Index; LVEF, Left Ventricular Ejection Fraction; BMI, Body Mass Index; CNY, China Yuan.

Among caregivers, there were 98 male caregivers (39.2%) and 152 female caregivers (60.8%). The average age was 60.20 + 13.31 years. Most of the caregivers are married (96.0%), and low level of education (70.4%). The most type of dyadic relationship between patient and caregiver is mostly spouse (54.8%). 64.8% of caregivers are employed. Most caregivers have moderate incomes. The majority of the caregivers spent less than one year providing care and more than two hours per day. 15.6% of caregivers had hypertension.

### Mutuality and depression scores in patients with chronic heart failure and their caregivers

The Table 2 and the radar map (Supplementary Figure 1) showed that CHF patients total score of mutuality and the scores of mutuality dimensions were higher than those of caregivers. It indicated that better mutuality perceived by patients. Compared with caregivers, CHF patients had significantly higher depression scores. It is also clear from the radar map that the depression of the patients was worse than that of the caregivers.

**TABLE 2:** Comparisons of mutuality scale scores and SDS scores between patients and caregivers (*n* = 250 dyads).

Characteristics	Patients (M ± SD)	Caregivers (M ± SD)	*t*	*P*
Love and affection	3.13 ± 0.68	2.96 ± 0.72	3.97	<0.001
Pleasurable activities	2.92 ± 0.73	2.69 ± 0.70	5.34	<0.001
Shared values	2.55 ± 0.86	2.27 ± 0.79	5.44	<0.001
Reciprocity	2.92 ± 0.71	2.56 ± 0.73	8.04	<0.001
Mutuality (total score)	2.91 ± 0.68	2.64 ± 0.67	6.99	<0.001
SDS	43.17 ± 13.65	32.00 ± 9.65	12.06	<0.001

SDS, Self-Rating Depression Scale.

Table 3 shows the relationship between mutuality dimensions and depression. Overall and all dimensions of mutuality were associated with depression. Caregivers’ overall mutuality and some mutuality dimensions were weakly associated with depression. Patients’ overall mutuality and mutuality dimensions were not associated with caregivers’ depression. Caregivers’ “love and affection” was associated with patients’ depression.

**TABLE 3:** Correlations of mutuality scale scores and SDS scores between patients and caregivers (*n* = 250 dyads).

		Patients						Caregivers					
		1	2	3	4	5	6	1	2	3	4	5	6
Patients	1	1											
	2	0.785**	1										
	3	0.661**	0.796**	1									
	4	0.807**	0.885**	0.806**	1								
	5	0.875**	0.949**	0.867**	0.970**	1							
	6	−0.389**	−0.361**	−0.391**	−0.416**	−0.422**	1						
Caregivers	1	0.527**	0.445**	0.349**	0.472**	0.490**	−0.132*	1					
	2	0.486**	0.550**	0.478**	0.509**	0.549**	–0.064	0.783**	1				
	3	0.397**	0.455**	0.497**	0.442**	0.479**	–0.076	0.587**	0.799**	1			
	4	0.516**	0.529**	0.512**	0.523**	0.561**	–0.114	0.759**	0.899**	0.826**	1		
	5	0.532**	0.547**	0.505**	0.537**	0.573**	–0.107	0.850**	0.957**	0.859**	0.971**	1	
	6	–0.108	–0.041	–0.088	–0.123	–0.100	0.247**	–0.085	–0.099	−0.153*	−0.142*	−0.131*	1

**P < 0.01, *P < 0.05; 1 = love and affection, 2 = pleasurable activities, 3 = shared values, 4 = reciprocity, 5 = mutuality total score, 6 = SDS, Self-Rating Depression Scale.

### Actor–partner effects of mutuality on depression

The results (Table 4 and Supplementary Figure 2) showed that there was an actor effect of CHF patients’ mutuality total score on depression (*B* = −0.536, *P*<0.001). Caregivers’ mutuality total score had a significant partner effect on patients’ depression (*B* = 0.200, *P* = 0.004). However, there were no partner effect of patients’ mutuality total score on caregivers’ depression and no actor effect of caregivers’ mutuality total score on depression.

**TABLE 4:** Actor and partner effects of mutuality on depression (*n* = 250 dyads).

	Actor and partner effects	Depression		
		Estimate	*B*	*P*
Mutuality (total score)	Actor: patient	–10.805	–0.536	<0.001
	Partner: patient	–0.530	–0.037	0.628
	Actor: caregiver	–1.566	–0.109	0.154
	Partner: caregiver	4.053	0.200	0.004
Love and affection	Actor: patient	–8.833	–0.441	<0.001
	Partner: patient	–1.240	–0.088	0.237
	Actor: caregiver	–0.517	–0.039	0.601
	Partner: caregiver	1.888	0.100	0.143
Pleasurable activities	Actor: patient	–8.743	–0.467	<0.001
	Partner: patient	0.247	0.019	0.805
	Actor: caregiver	–1.508	–0.109	0.148
	Partner: caregiver	3.762	0.193	0.006
Shared values	Actor: patient	–7.400	–0.468	<0.001
	Partner: patient	–0.174	–0.016	0.829
	Actor: caregiver	–1.779	–0.145	0.044
	Partner: caregiver	2.702	0.156	0.019
Reciprocity	Actor: patient	–9.489	–0.490	<0.001
	Partner: patient	–0.924	–0.067	0.359
	Actor: caregiver	–1.417	–0.107	0.147
	Partner: caregiver	2.675	0.142	0.034

Table 4 and Supplementary Figure 3 showed the actor-partner effect of mutuality dimensions on depression. We found that patients’ “love and affection” had an actor effect on depression (*B* = −0.441, *P* < 0.001). There were an actor effect of patients’ “pleasurable activities” on depression (*B* = −0.467, *P* < 0.001) and a partner effect of caregivers’ “pleasurable activities” on patients’ depression (*B* = 0.193, *P* = 0.006). Patients’ “shared values” (*B* = −0.468, *P* < 0.001) and caregivers’ “shared values” (*B* = 0.156, *P* = 0.019) influenced patients’ depression. We also found an actor effect of caregivers’ “shared values” on caregivers’ depression (*B* = −0.145, *P* = 0.044). In addition, the lower the patient “reciprocity” score (*B* = −0.490, *P* < 0.001) and the higher the caregiver “reciprocity” score (*B* = 0.142, *P* = 0.034), the lower the patient’s depression score.

## Discussion

The purpose of this study was to evaluate the relationship between depression and mutuality in patients with CHF and their family caregivers. Our main finding was that higher patient mutuality and lower caregiver mutuality were associated with decreased patient depression, but we didn’t find these associations in the caregivers. To our knowledge, this is an earlier study in China to explore the relationship between mutuality and depression in patients with CHF and their caregivers in China using an APIM.

The results showed that mutuality scores of patients and caregivers were 2.91 ± 0.68 and 2.64 ± 0.67. The patient-caregiver mutuality was at a moderate level in our study. The level of mutuality of patients was higher than that of caregivers, which was consistent with the results reported by Hooker et al. (2018), who studied 99 dyads of HF patients and their caregivers. The caregivers’ mutuality score in our study was higher than that of caregivers of stroke (Pan et al., 2017) and pancreatic cancer patients (Zhang et al., 2020). This difference may be due to the severity of stroke and pancreatic cancer, which can affect communication between caregiver and patient and which requires complex and difficult tasks that may affect the caregivers’ mutuality.

Depression is a common psychological symptom of patients with CHF. It leads to adverse problems such as increased level of cardiac function and decreased functional ability of CHF patients (Lossnitzer et al., 2020). The depression scores of patients with chronic heart failure in this study were higher than those of previous studies (Chung et al., 2016; Matsuda et al., 2021). The possible reasons are as follows: (1) In this study, 81.6% of elderly patients with chronic heart failure had higher NYHA class. The complexity of the disease and the high cost of medical treatment led to many patients have negative emotions. (2) Differences in depression evaluation methods adopted by researchers. The survey tool used in this study is the SDS, which has been used to assess emotional states in China for a long time. Some studies have also confirmed that the SDS has high specificity and can accurately reflect patients’ mood disorders (Zhang et al., 2008). Depression also causes burden on caregivers. In this study, we found that patients scored significantly higher for depression than caregivers. An Italian study of 366 patients with HF and their caregivers reported the same result (Dellafiore et al., 2019). Both patients and caregivers are affected by CHF, but patients tend to be much more affected than caregivers. As a result, their emotional health is adversely affected.

In the dyadic analysis, we found that patient mutuality influenced patient depression, which was consistent with Lyons et al. who found that better relationship quality was significantly associated with decreased depressive symptoms for patients (Lyons et al., 2020). More specifically, our study demonstrated that all the 4 dimensions of patients’ mutuality had negative influence on patients’ depression. Mutuality is an important factor in the treatment of patients with CHF. Patients with high level of mutuality are mostly able to actively cope with the disease, constantly adjust their psychological state, and reduce the generation of negative emotions (Peng et al., 2021).

In addition, we also found significant actor effect on caregivers. Caregivers’ “shared values” inversely influenced their depression. In other words, as their “shared values” increased their depression scores decreased. This is an important finding for caregivers because we did not find the effect of caregiver mutuality total scores on their depression. In other studies, mutuality was analyzed as a total score, making it difficult to see small effects. Through the analysis of caregiver mutuality dimensions, we found that “shared values” was an important factor. “Shared values” reflects the convergence of views between the patient and the caregiver on some issues. For caregivers, they hope to reach an agreement with patients when dealing with their disease problems, so as to avoid conflicts and negative emotions (Bouldin Aikens et al., 2019).

We found a partner effect of caregiver mutuality on patient depression. In other words, caregivers’ better mutuality was associated with patients’ greater depression. Specifically, caregiver higher scores in “pleasurable activities,” “shared values,” and “reciprocity” were associated with patient greater depression. The results were unexpected because it showed that caregiver mutuality did not improve patient depression. It’s hard to interpret these results. This may be because the patients’ disease condition in this study is very serious, and caregivers with a high level of mutuality care too much about the patients. Thus, increasing the patients’ disease treatment pressure and negative emotions. The effect of caregiver’s mutuality on the patient’s depression could be different depending on the level of mutuality. However, this relationship needs to be further studied.

To sum up, this study emphasizes the interdependence theory. This theory holds that there is strong interpersonal interaction between dyads with intimate relationship. Individual emotion, cognition or behavior are easily transferred between dyads, and may eventually affect the health outcome of the partner (Wickham and Knee, 2012). Our results also demonstrate the interaction between CHF patients and caregivers.

This study has several advantages and limitations. The study was based on analysis using the APIM, thus the interdependence of dyadic data was well controlled. We used radar maps to compare patient-caregiver dyads variables and it was able to see the difference visually. However, the sample size included in this study was small, and a larger sample is needed to verify our results. Finally, this study only included CHF patients from two hospitals in one city in China, thus the results should be cautiously generalized to other locations.

## Conclusion

We assessed the relationship between depression and mutuality in patients with CHF and caregivers in China. The level of mutuality and depression of patients was significantly higher than that of caregivers. Our results highlight the importance of evaluating mutuality in patients and caregivers in clinical settings to improve outcomes for the patient-caregiver dyads.

## Relevance for clinical practice

This study determined the relationship between depression and mutuality in patients with chronic heart failure and their family caregivers. The results of this study indicate the importance of evaluating the relationship between patients and caregivers. However, caregiver’s physical and mental health is often ignored by medical staff in clinical settings. In most cases, patients view intimate family caregivers as the most important emotional and support resources in their lives. When the primary family caregiver has negative emotions, they provide less care to the patient than usual. This will lead to a decrease in the patient’s perceived emotions and support. Thus, the assessment of mutuality in patients and caregivers should be strengthened in clinical settings, as it is an important factor in improving the adverse mood of family caregivers.

## Data availability statement

The raw data supporting the conclusions of this article will be made available by the authors, without undue reservation.

## Author contributions

TZ was responsible for drafting the manuscript, as well as the acquisition, analysis, and interpretation of data. JQ collected, analyzed, and interpreted the data. HS and MX collected the data. YL provided professional advice and performed much of the editing of the manuscript. All authors read and approved the final manuscript.

## Supplementary material

Supplementary Figure 1 Comparisons of mutuality scale scores and SDS scores between patients and caregivers.

Supplementary Figure 2 Actor and partner effects of CHF patient’s mutuality and caregiver’s mutuality on depression. ^**^*P* < 0.01; ^***^*P* < 0.001.

Supplementary Figure 3 Actor and partner effects of CHF patient’s mutuality dimensions and caregiver’s mutuality dimensions on depression. **P* < 0.05; ^**^*P* < 0.01; ^***^*P* < 0.001.
