# Supplementary figures and images for: Relationship between mutuality and depression in patients with chronic heart failure and caregivers in China: An actor-partner interdependence model analysis
Source: Front Psychol. 2022 Sep 8;13:928311. doi: 10.3389/fpsyg.2022.928311 (PMC9493196; doi:10.3389/fpsyg.2022.928311)

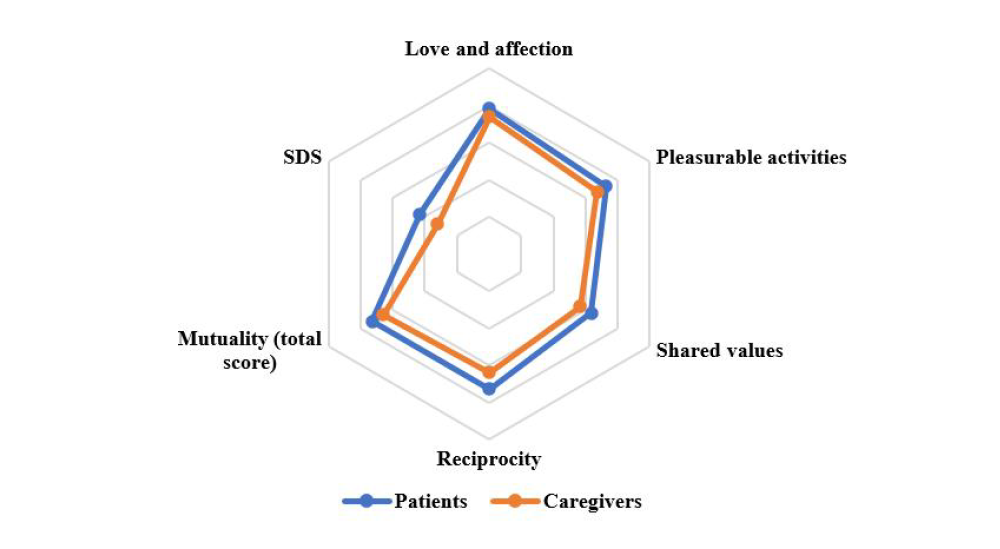

Supplement: Supplementary Figure 1 — Comparisons of mutuality scale scores and SDS scores between patients and caregivers. [file Image_1.tif]

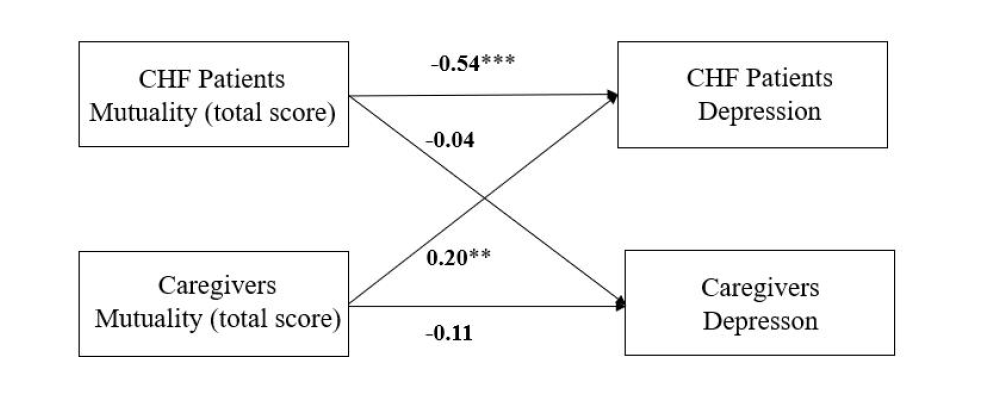

Supplement: Supplementary Figure 2 — Actor and partner effects of CHF patient’s mutuality and caregiver’s mutuality on depression. **P < 0.01; ***P < 0.001. [file Image_2.tif]

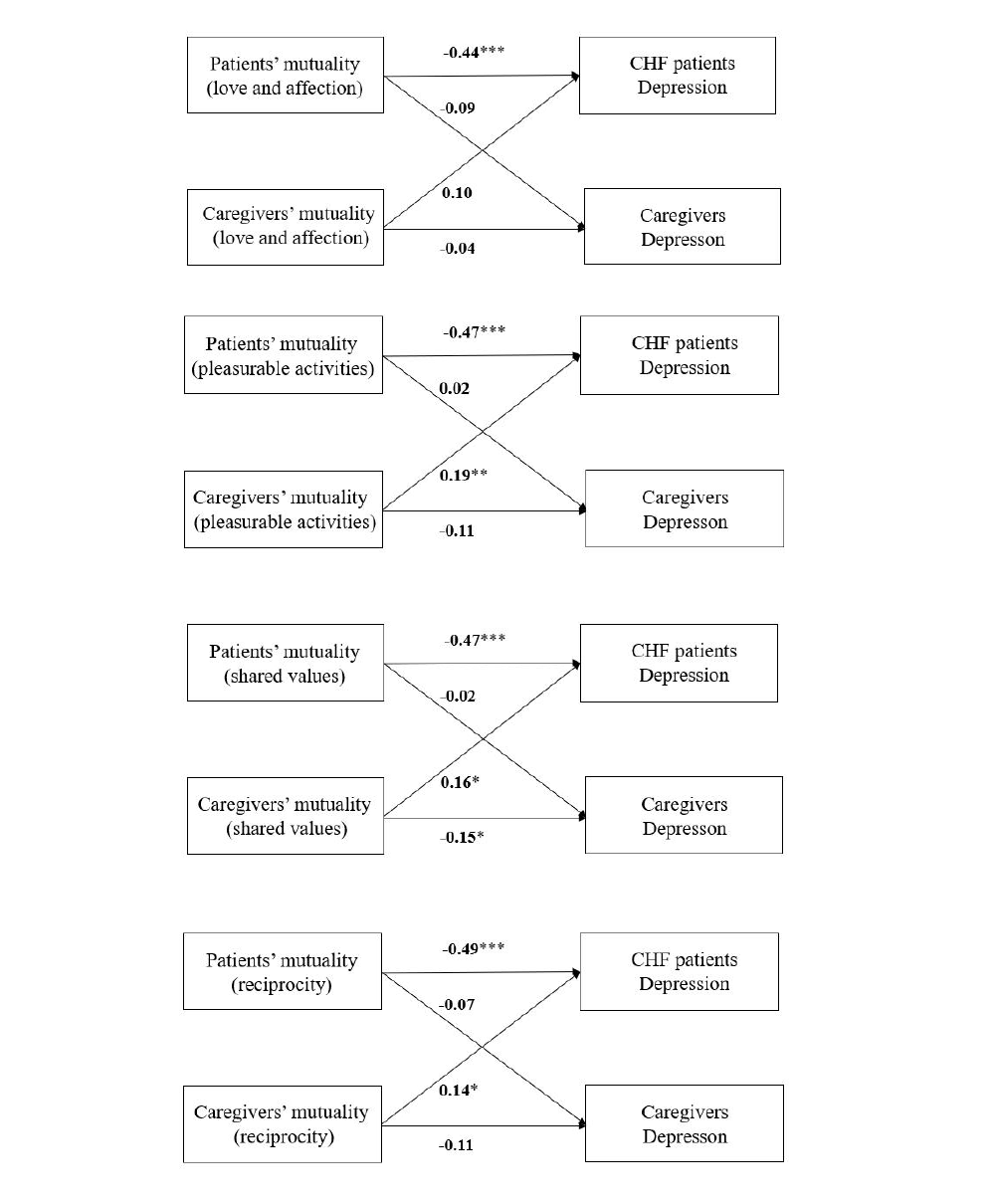

Supplement: Supplementary Figure 3 — Actor and partner effects of CHF patient’s mutuality dimensions and caregiver’s mutuality dimensions on depression. *P < 0.05; **P < 0.01; ***P < 0.001. [file Image_3.tif]
